# Transposons play an important role in the evolution and diversification of centromeres among closely related species

**DOI:** 10.3389/fpls.2015.00216

**Published:** 2015-04-07

**Authors:** Dongying Gao, Ning Jiang, Rod A. Wing, Jiming Jiang, Scott A. Jackson

**Affiliations:** ^1^Center for Applied Genetic Technologies, University of GeorgiaAthens, GA, USA; ^2^Department of Horticulture, Michigan State UniversityEast Lansing, MI, USA; ^3^Department of Plant Sciences, Arizona Genome Institute, University of ArizonaTucson, AZ, USA; ^4^Department of Horticulture, University of Wisconsin-MadisonMadison, WI, USA

**Keywords:** centromere, rice, transposon, comparative genomics, nested insertions

## Abstract

Centromeres are important chromosomal regions necessary for eukaryotic cell segregation and replication. Due to high amounts of tandem repeats and transposons, centromeres have been difficult to sequence in most multicellular organisms, thus their sequence structure and evolution are poorly understood. In this study, we analyzed transposons in the centromere 8 (*Cen8*) from the African cultivated rice (*O. glaberrima*) and two subspecies of the Asian cultivated rice (*O. sativa*), *indica* and *japonica*. We detected much higher transposon contents (>69%) in centromere regions than in the whole genomes of *O. sativa* ssp. *japonica* and *O. glaberrima* (~35%). We compared the three *Cen8s* and identified numerous recent insertions of transposons that were frequently organized into multiple-layer nested blocks, similar to nested transposons in maize. Except for the Hopi retrotransposon, all LTR retrotransposons were shared but exhibit different abundances amongst the three *Cen8s*. Even though a majority of the transposons were located in intergenic regions, some gene-related transposons were found and may be involved in gene diversification. Chromatin immunoprecipitated (ChIP) data analysis revealed that 165 families from both Class I and Class II transposons were found in CENH3-associated chromatin sequences. These results indicate essential roles for transposons in centromeres and that the rapid divergence of the *Cen8* sequences between the two cultivated rice species was primarily caused by recent transposon insertions.

## Introduction

Centromere serves essential functions in the faithful segregation and transmission of chromosome during eukaryotic cell division (Henikoff et al., 2001). The centromere of *S. cerevisiae* is comprised of a 125-bp DNA sequence, called a “point” centromere (Carbon and Clarke, 1990). However, centromeres of most multicellular eukaryotes consist of large amounts of highly repetitive sequences including satellite tandem repeats and transposable elements (TEs) that are organized into large repeats blocks (Zhong et al., 2002). Due to their highly repetitive nature, centromeres are difficult to sequence and assemble. For example, whole genome sequences are available for many eukaryotes including model organisms such as human, *D. melanogaster* and *A. thaliana*; however, none of the centromeres in these model eukaryotes have been completely sequenced (Copenhaver et al., 1999; Schueler et al., 2001; Sun et al., 2003). Thus, many details on centromere composition and organization remain to be discovered.

Unlike many eukaryotic centromeres that contain megabase-sized (Mb)-repeats, centromere 8 (*Cen8*) in rice variety Nipponbare (*O. sativa* L. ssp. *japonica*) harbors only ~65-kilobases (kb) of tandem repeats (Cheng et al., 2002) and was the first completely sequenced centromere in higher organisms. The *Cen8* contains not only the 155-bp CentO tandem repeats and transposons but also expressed genes that led to the hypothesis that the rice *Cen8* may represent a recently formed centromere (Nagaki et al., 2004; Wu et al., 2004). The centromeres 4 and 3 from Nipponbare were also completely sequenced and were similar to the scenario described for *Cen8* (Zhang et al., 2004; Yan et al., 2006). Recently, the *Cen8s* from another rice variety Kasalath (*O. sativa* L. *ssp. indica*), *O. brachyantha* and *O. glaberrima* were analyzed and colinearity and conservation of centromeric genes were observed in these orthologous centromeres (Gao et al., 2009; Wu et al., 2009; Fan et al., 2011). Therefore, *Cen8* offers an opportunity to investigate centromere evolution in eukaryotic organisms.

Transposons are repetitive DNA sequences that have the capability to move (transpose) from one location to another in genome. Transposon movement can result in mutations, alter gene expression, induce chromosome rearrangements and, due to increase in copy numbers, enlarge genome sizes. Thus, they are considered an important contributor for gene and genome evolution (Kazazian, 2004). Transposons represent the most abundant repeats in most plant genomes. For example, these elements constitute more than 85% of the maize (*Z. may*) genome (Schnable et al., 2009). Some transposons are located in genic regions, but most are found in heterochromatic regions including telomeres and centromeres. Previous studies have shown that transposons serve as essential components for functional centromeres (Nagaki et al., 2004) and for maintaining centromeric and telomeric stability and heterochromatic silencing (Maxwell et al., 2006; Zaratiegui et al., 2011). Additionally, transposons have been domesticated by a host genome to facilitate centromere formation (Cam et al., 2008). Transposons are often transcriptionally inactive and heavily methylated in centromeric regions as their activity can be deleterious to the host genome. For example, reactivation of retrotransposons can cause meiotic failure in spermatocytes in *M. musculus* (Bourchis and Bestor, 2004), and impair centromere function resulting in lagging chromosomes in *S. pombe* (Volpe et al., 2002).

To gain more insight into centromeric transposons and their role in centromere evolution, we analyzed the composition and organization of transposons in three orthologous *Cen8* sequences—African cultivated rice (*O. glaberrima*) and two subspecies of Asian cultivated rice (*O. sativa*), *indica* and *japonica*. We identified numerous recent transposon insertions, including some into centromeric genes, and found that the transposons were often organized into nested blocks. We also searched the ChIP cloning data and identified CENH3-associated transposons. Our results reveal the highly dynamic nature of transposons in the *Cen8* region and suggest that transposons played a pivotal role in the rapid divergence of the *Cen8* DNA sequences between these two cultivated rice species.

## Materials and methods

### Plant materials

A total of eight *Oryza* species were used in this study, including Nipponbare (*Oryza sativa* L. ssp. *japonica*, AA), African cultivated rice (*O. glaberrima*, AA) and six wild rice species: *O. nivara* (AA), *O. longistaminata* (AA), *O. rufipogon* (AA), *O. punctata* (BB), *O. minuta* (BBCC), and *O. officinalis* (CC). The seeds from all eight rice species were planted and grown in the greenhouse, and young leaves were collected to extract DNA using the cationic detergent cetyl-trimethylammonium bromide (CTAB) method.

### Centromere 8 sequences

The 1.3-Mb *Cen8* sequence of *O. glaberrima* was sequenced by the Evolutionary Genomics of a Rice Centromere Project (Fan et al., 2011), the sequence assembly was validated by fluorescence *in situ* hybridization (FISH), Fiber-FISH and PCR analyses. The *Cen8* from Kasalath was obtained from the rice genome research program (RGP) website (http://rgp.dna.affrc.go.jp/E/Publicdata.html). The 2.4-Mb *Cen8* of Nipponbare was downloaded from the GenBank and its boundary was determined based on the previous researches (Nagaki et al., 2004; Wu et al., 2004, 2009; Yan et al., 2005). The corresponding locations of three *Cen8s* in the rice chromosome 8 pseudomolecule (accession no. AP008214) are shown in Supplementary Figure 1.

### Annotation of transposons and sequence alignment

In order to annotate TEs in the *Cen8s*, the rice transposon library (Ning Jiang, Mich. State Univ., personal communication) was incorporated with other published transposon databases (Nagaki et al., 2005; Chaparro et al., 2007), and used as a TE library to screen the sequences using RepeatMasker (http://www.repeatmasker.org). The program was run with default settings and “nolow” option to avoid masking the low complexity DNA or simple repeats. In addition, we also set a cutoff score greater than 300 and hit sequence larger than 50 bp in length. All reads obtained by RepeatMasker were inspected manually to (1) determine the exact boundaries of each element and their target site duplications (TSD), (2) remove the overlap regions which were annotated as different transposons, (3) determine the elements that were nested by other transposons or other copies of itself, and (4) count the copy number of transposons in *Cen8* sequences.

To track transposon dynamics and to detect newly inserted transposons, we compared the *Cen8* sequences from two cultivated rice species. The orthologous transposons were defined by a combination of three approaches: (1) structural futures of transposons including long terminal repeats (LTRs), terminal inverted repeats (TIRs), and target site duplications (TSDs); (2) sequence alignment; and (3) the centromeric genes flanking the TEs.

The sequence alignments were conducted using the Artemis Comparison Tool (ACT, https://www.sanger.ac.uk/resources/software/act). The three *Cen8s* were first used for all-against-all BLASTN searches with the -m 8 option between each other. The output files and the *Cen8s* were then used to generate sequence alignment with ACT using the default options.

### DNA hybridization

DNA hybridization was performed with 10 μg of total DNA digested with *EcoR* I (Invitrogen, Carlsbad, CA). The digested DNA fragments were separated by electrophoresis on a 1.0% (w/v) agarose gel at 55 v for 11 h and then transferred to a nylon membrane (GE Healthcare Life Sciences, Pittsburgh, PA). The LTRs of three retrotransposons were used to design primers to amplify DNA from Nipponbare. The primers used were as follows: *CRR1* (Forward, 5′-GCAAGGACCAATGACTAGAG-3′; Reverse, 5′-CAAGCAAGAACAAGTTGACA-3′); *RIRE3* (Forward, 5′- GTGCATGGTTTTGATAGTAGC-3′; Reverse, 5′-GGTGTACATCTTTACCCACAA -3′) and *Hopi* (Forward, 5′-TAGAGACTTGAGGCAGACACG -3′; Reverse, 5′- GTCACAAATCGGTCATTCTTG-3′). The PCR products were labeled with [α−^32^P]-dCTP using the rediprime II random prime labeling system (GE Healthcare Life Sciences, Pittsburgh, PA) according to the manufactures instructions. Blots were hybridized at 58.5°C for overnight and washed with 1.5× SSC solution for 30 min and 1× SSC for 30 min. The membrane was exposed on a Fuji-image plate and the hybridization signals were captured using a Fujifilm FLA-5100 multifunctional scanner.

## Results

### Transposon abundance in three *CEN8s*

A comprehensive TE library was used to annotate transposons in the *Cen8* sequences from Nipponbare (Nagaki et al., 2004; Wu et al., 2004), Kasalath (Wu et al., 2009) and *O. glaberrima* (Fan et al., 2011). Both RNA retrotransposons (Class I) and DNA transposons (Class II) were identified, but contributed different fractions in the three *Cen8s*.

In *Cen8* of Nipponbare, a total of 858 TEs were identified that make up 69.2% of the sequence (Table 1), this fraction is much higher than that the 51% found in centromere 3 (*Cen3*) (Yan et al., 2006) or the 35% in the whole genome sequence (International Rice Genome Sequencing Project, 2005), indicating higher transposon activity and/or retention in *Cen8*. There were more Class II transposons than Class I elements (492 vs. 366), however, the Class I transposons contributed much more sequence, 57.5% vs. 11.7%, due to larger average size of Class I elements. Ty3-gypsy elements were the most abundant LTR-retrotransposons contributing more than half of the *Cen8* sequence. We detected DNA transposons from five superfamilies in the *Cen8* including Mutator, hAT, CACTA, PIF/Harbinger, and Helitron (Table 1). Helitron transposons have not been previously described in rice centromeres (Nagaki et al., 2004; Wu et al., 2004; Zhang et al., 2004; Yan et al., 2006).

**Table 1:** **Summary of transposons in 3 *Cen8* sequences**.

**Genomes**	**Nipponbare**			***O. glaberrima***			**Kasalath**		
	**Copy no**.	**Coverage (bp)**	**Content (%)**	**Copy no**.	**Coverage (bp)**	**Content (%)**	**Copy no**.	**Coverage (bp)**	**Content (%)**
**CLASS I**									
*Ty1-copia*	33	73,608	3.04	19	47,671	3.63	32	78,959	3.51
*Ty3-gypsy*	322	1,313,356	54.20	209	736,593	56.07	293	1,296,074	57.62
Other	11	6278	0.26	6	3,918	0.30	3	1009	0.04
Total Class I	366	1,393,242	57.50	234	788,182	60.00	328	1,376,042	61.17
**CLASS II**									
hAT	47	39,572	1.63	16	6149	0.47	34	28,951	1.29
CACTA	32	66,688	2.75	9	34,971	2.66	24	58,694	2.61
Mutator	121	82,342	3.40	59	34,986	2.66	96	65,284	2.90
Harbinger	23	19,643	0.81	14	21,941	1.67	16	17,181	0.76
Helitron	50	27,835	1.15	15	9319	0.71	39	31,267	1.39
MITE/Stow	84	16,554	0.68	36	6798	0.52	65	13,302	0.59
MITE/Tourist	134	29,745	1.23	48	9753	0.74	104	23,801	1.06
Other	1	111	0.005	1	111	0.01	1	111	0.005
Total Class II	492	282,490	11.66	198	124,028	9.44	379	238,591	10.61
Total TEs	858	1,675,732	69.15	432	912,210	69.44	707	1,614,633	71.78

LTR retrotransposons, but not DNA transposons, were previously annotated for *Cen8* of Kasalath (Wu et al., 2009). Our TE library recognized 707 TEs in Kasalath *Cen8*, including 379 DNA transposons and 328 LTR retroelements. These TEs account for 71.8% of the centromere sequence (Table 1). In addition to the previous study that identified 222 LTR retrotransposons covering 1,241,769 bp (Wu et al., 2009), we identified DNA elements and larger number and higher coverage of LTR retrotransposons in the Kasaltah *Cen8*.

In *Cen8* of *O. glaberrima*, 432 TEs were detected that contributed 69.4% of the sequence. The TE content in the *Cen8* was similar to that in Nipponbare but a little less than Kasalath (Table 1). However, the fraction was twice as much as the genome average for *O. glaberrima* (34.3%, Wang et al., 2014), again indicating a higher TE abundance in the *Cen8* region.

### Compositions of LTR retrotransposons in three *CEN8s*

As LTR retrotransposons contribute a significant fraction of the *Cen8s*, we further analyzed the coverage and copy numbers of different retrotransposon families. We manually inspected the sequence annotation and identified intact elements and/or intact solo-LTRs for 44 families of LTR retrotransposons in *Cen8* from Nipponbare. These 44 families were the focus of the following comparisons and other LTR retrotransposons were excluded. We identified all 44 retrotransposon families in Kasalath's *Cen8*. However, different families show distinct fractions between the *Cen8* sequences. 15 families were more prevalent than others, contributing 45 and 49% of *Cen8* from Nipponbare and Kasalath, respectively (Supplementary Table 1), or 79 and 86% of the total LTR retrotransposons in the *Cen8s*, respectively. Further comparisons indicated that all 15 retrotransposon families but *Hopi* were also present in *Cen8* of *O. glaberrima*. Among the 15 families, *RIRE3* was the most dominant family constituting 10–14.6% of three *Cen8*s, and highest coverage of *RIRE3* was found in Kasalath's *Cen8*. The centromere retrotransposons of rice (*CRR*) was highly conserved between rice, maize, and other grasses and likely plays important role for functional centromeres (Jiang et al., 2003; Nagaki et al., 2004). We identified *CRRs* in the *Cen8*s from both *O. sativa* and *O. glaberrima*, but lower coverage and fraction of *CRRs* were detected in *Cen8* of *O. glaberrima* (Supplementary Table 1). These results suggest distinct amplification dynamics between different retrotransposon families in these three rice genomes.

**Figure 1:**
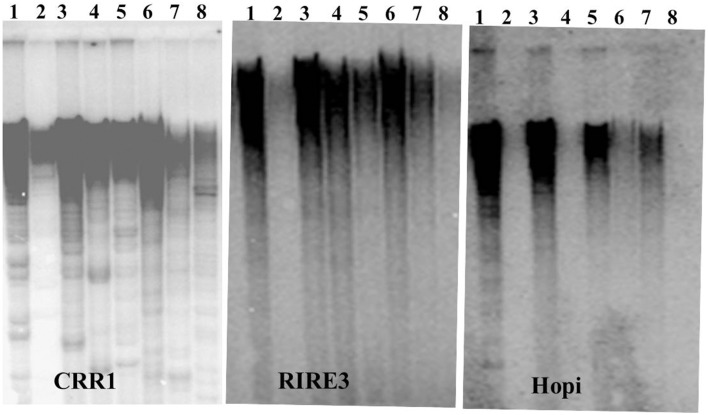
**Southern blots analysis of three LTR retrotransposons**. 1. *O. sativa*, 2. *O. glaberrima*, 3. *O. nivara*, 4. *O. longistaminata*, 5. *O. rufipogon*, 6. *O. minuta*, 7. *O. punctata*, 8. *O. officinalis*.

We next conducted Southern blot with the LTRs of *CRR*, *RIRE3*, and *Hopi* to gain insights into the genomic abundance of these retrotransposons. Strong hybridization signals of *RIRE3* and *CRR* were detected in *O. sativa* and weak signals were detected in *O. glaberrima* (Figure 1). This suggests that there was higher amplification activity of *RIRE3* and *CRR* in *O. sativa* or that the mobility of the retrotransposons was suppressed in *O. glaberrima*. Using the *Hopi* probe, very weak hybridization signals were detected in *O. glaberrima* and *O. longistaminata* but strong signals were found in *O. sativa*, *O. nivara*, *O. rufipogon*, and *O. punctata* (Figure 1), indicating that *Hopi* elements were likely removed or diverged in *O. glaberrima* after the split from *O. punctata*. Therefore, our Southern blots are consistent with the comparative analyses and indicate differential dynamics of the three retrotransposon families between *O. sativa* and *O. glaberrima*.

### Nested organizations of transposons in three *CEN8s*

In organisms, such as maize, with high quantities of TEs, transposons are frequently clustered and/or nested which is useful to track the evolutionary history of transposons and reconstruct insertion patterns (Kronmiller and Wise, 2008). Nested TEs are not very common in euchromatic regions of rice and *A. thaliana* (Du et al., 2006; Kronmiller and Wise, 2008). However, the TEs in *Cen8*s are organized similarly to that seen in maize as we found numerous nested transposon blocks in which a host transposon contains one or multiple elements from the same or other families.

**Figure 2:**
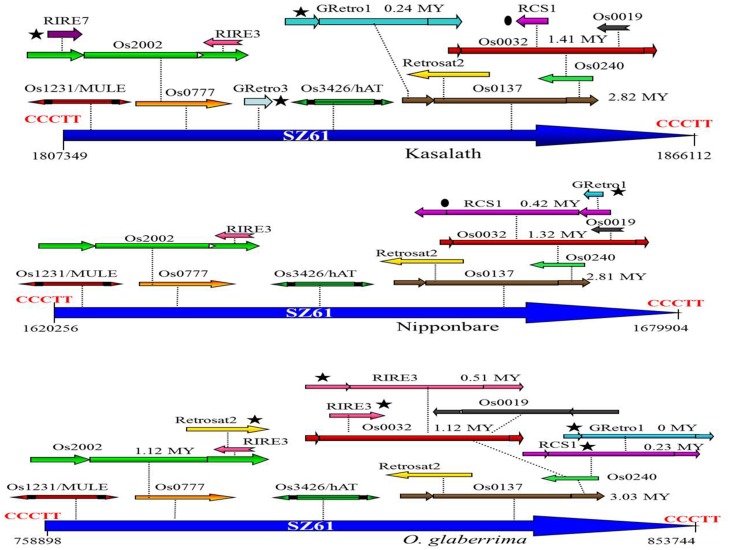
**Comparison of an orthologous transposon nested block among three *Cen8*s**. A solo-LTR of SZ61 retrotransposons is flanked by 5-bp TSD (CCCTT) and serves as the host element for other transposons. The ovals indicate a shared transposon between the *Cen8*s of Nipponbare and Kasalath and the stars indicate lineage-specific transposons. The insertion times for each of complete LTR retrotransposons are notated near the retroelement.

A total of 57 nested TE blocks were identified in the *Cen8* of Nipponbare, ranging in size from 409 to 82,419 bp with an average size of 16,624 bp. 26 and 40 nested blocks were found in the *Cen8* of *O. glaberrima* and Kasalath with sizes varying from 1022 to 94847 bp (18,803 bp average) and 409 to 129,488 bp (22,669 bp average), respectively. The TEs in nested blocks accounted for 37.2, 39.1, and 40.3% of the *Cen8* in *O. glaberrima*, Nipponbare, and Kasalath, respectively. Interestingly, some TEs were involved in multi-layered nested blocks in which previous nested elements further served as hosts for additional TEs. For instance, we found a solo LTR of *SZ61* that harbors two DNA transposons (*Os1231* and *Os3426*) and 10 LTR retrotransposons that were organized into two to six-layer nested blocks in Nipponbare. The orthologous solo LTR also hosts 14 and 15 transposons in Kasalath and *O. glaberrima*, respectively (Figure 2). The size of the orthologous nested block was 59.6, 58.8, and 94.8 Kb in Nipponbare, Kasalath and *O. glaberrima*, respectively, suggesting a recent expansion in *O. glaberrima*.

To determine if different TE classes exhibited insertion preferences, all nested transposons were divided into four groups based on host and nested transposons: RNA TEs inserted into RNA TEs, RNA TEs inserted into DNA TEs, DNA TEs inserted into RNA TEs and DNA TEs inserted into DNA TEs. Among 142 nested TEs (106 retrotransposons and 36 DNA transposons) in *Cen8* of Nipponbare, 99 retrotransposons were located in other LTR retroelements, only seven retrotransposons inserted into DNA elements, a ratio of 14.1 (99/7). However, the numbers of DNA transposons that inserted into RNA and DNA elements were similar (19 vs. 17) (Supplemental Table 2). Given that the ratio of number and coverage of RNA to DNA transposons was 0.74 (366/492) and 4.93 (1,393,242 bp/282,490 bp), these results suggest that LTR-retroelements are frequently inserted into other LTR families or other copies of itself. A similar trend was also detected in the *Cen8s* from Kasalath and *O. glaberrima* as 75.9% (107/141) and 76.3% (71/93) of the total nested TEs were into the group of RNA into RNA, respectively.

### CENH3-associated transposons

**Figure 3:**
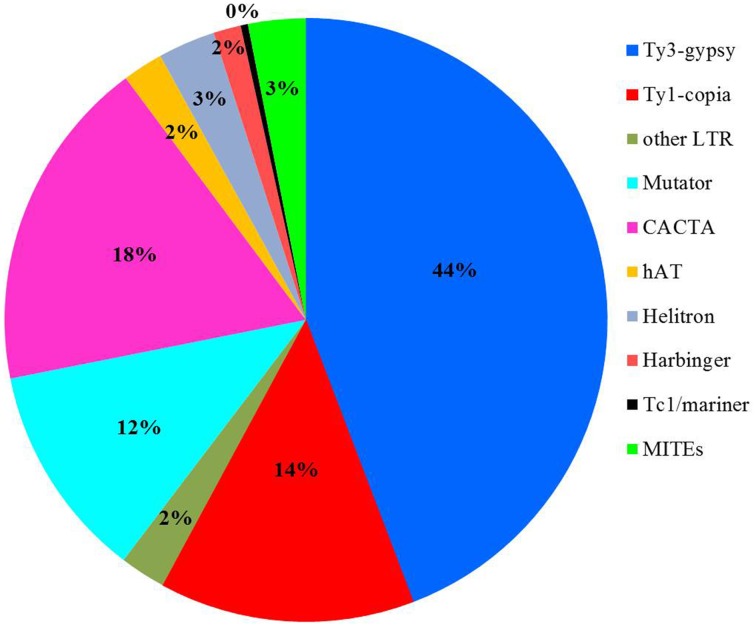
**Summary of CENH3-assocated transposons in Nipponbare**.

To detect if transposons can bind to the centromere-specific histone and to serve as component for centromere formation, the sequencing data obtained from chromatin immunoprecipitated DNA with an anti-CENH3 antibody in rice (Zhang et al., 2013) was used as query to search against the transposon library. 8,883,768 sequences or 7.8% of the total reads showed significant sequence similarity to rice transposons (*E*-value < 1 × 10^−5^). Further investigation indicated that 60% of the 8,883,768 CENH3-associated chromatin sequences were related to LTR retrotransposons, and the other 40% of the reads shared significant sequence similarity to different superfamilies of DNA transposons, including Mutator, CACTA, hAT, Helitron, Harbinger, and Tc1/mariner (Figure 3). We found that 165 transposon families, 77 retrotransposons and 88 DNA transposons, were targeted by more than 5000 reads (Supplemental Table 3). Some transposon families were targeted by extremely numerous reads. For instance, 473,463 reads exhibited significant sequence similarity to *CRR* transposons supporting an essential role of *CRRs* in formation of functional centromere (Nagaki et al., 2004). Interestingly, 609,689 and 520,971 reads shared sequence similarity to *RIRE3* and *RIRE8*, respectively indicating that these two retrotransposon families are likely involved in interaction with the centromere specific H3 histone variant (CENH3).

**Figure 4:**
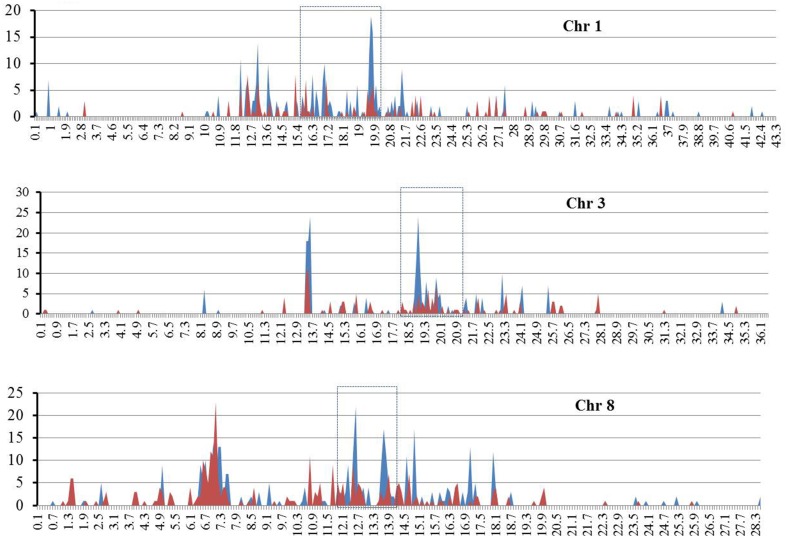
**The distributions of RIRE3 and RIRE8 retrotransposons in three rice chromosomes**. The X axis indicates the location of retrotransposons on chromosome (Mb) and Y axis means the copy number of retrotransposonsin 100-kb window. Blue and red line represents RIRE3 and RIRE8, respectively. Centromere locations are marked by boxes with broken lines.

Given that both *RIRE3* and *RIRE8* are enriched in *Cen8* of Nipponbare, 10.0% and 5.1% of the total sequence, respectively (Supplementary Table 1), we hypothesized that they were likely abundant throughout rice genome. To test this hypothesis and to determine the genome distribution of the two retrotransposon families, the combined TE library was used to screen the entire rice genome sequence (International Rice Genome Sequencing Project, 2005), 4098 *RIRE3* elements and 2087 *RIRE8* elements were detected that contributed 2.0 and 1.5% of the rice genome, respectively. This result shows the prevalence of the two retrotransposons in the rice genome, however, the distributions were uneven as *RIRE3* and *RIRE8* were much more abundant in *Cen8* than in the rice genome as a whole. The genomic distributions of *RIRE3* and *RIRE8* revealed that they dispersed throughout the rice genome but that centromeric and pericentromeric regions had much higher densities than any other locations across the 12 rice chromosomes (e.g., Figure 4—distributions of *RIRE3* and *RIRE8* on chromosomes 1, 3, and 8).

### Contribution of transposons to annotated centromere genes

Previous studies identified expressed genes in centromeres from both cultivated and wild rice species (Nagaki et al., 2004; Wu et al., 2004, 2009; Yan et al., 2006; Fan et al., 2011). Since ~70% of *Cen8s* sequences consist of TEs, we wanted to determine if TEs were involved in the formation of centromeric genes. Based on the Rice Genome Annotation Project Data (http://rice.plantbiology.msu.edu), 106 genes that do not encode transposon proteins were found in the *Cen8* of Nipponbare. We analyzed the 106 centromere genes and identified 28 that contain transposon-related sequences. A total of 53 transposon sequences were detected including 47 complete elements and five complete solo-LTRs. Further investigation indicated that 69.8% (37/53) of the transposons were located in introns of the genes, and another 30% served as coding DNA sequences (CDSs) or untranslated regions (UTRs) (Table 2). Among the 53 transposons, 75.5% (40/53) were from MITEs or MULEs, consistent with previous study that showed that MITEs and MULEs were enriched in genic regions (Feschotte and Pritham, 2007). However, only six retrotransposons were found in the centromere genes even though retrotransposons contributed 57.5% of the *Cen8* sequence. In the *Cen8s* of Kasalath and *O. glaberrima*, 22 and 13 genes were found to harbor transposon sequences, most of these were MITEs or MULEs and show a similar trend as seen for *Cen8* of Nipponbare (Table 2). It is worth noting that some centromere genes contain more than two transposons. For example, there is one Mutator transposon and two MITEs in the first, fourteenth and sixteenth intron of an expressed centromere gene, *LOC_Os08g21590*, in Nipponbare encoding a phosphatidylinositol 3-kinase. Additionally, the 3′ UTR of the gene also contains a fragmented solo LTR (Supplemental Figure 2).

**Table 2:** **Summary of centromere genes contained transposons**.

**Super-family**	**Nipponbare**				**Kasalath**				***O. glaberrima***			
	**CDS**	**Intron**	**UTR**	**all**	**CDS**	**Intron**	**UTR**	**all**	**CDS**	**Intron**	**UTR**	**all**
*Ty3-Gypsy*	5	0	0	5	2	1	0	3	2	0	0	2
*Ty1-Copia*	0	1	0	1	0	1	0	1	1	0	0	1
hAT	0	2	0	2	0	2	0	2	0	1	0	1
MULEs	4	5	1	10	4	4	1	9	2	2	1	5
Helitron	0	2	0	2	0	2	0	2	0	2	0	2
PIF/Har-binger	1	1	1	3	1	0	1	2	0	0	1	1
MITEs	1	26	3	30	1	20	3	24	0	13	0	13
Total	11	37	5	53	8	30	5	43	5	18	2	25

### Comparison of transposons between three *CEN8s*

To analyze transposon dynamics during centromere evolution, we compared transposons in the three *Cen8s*. All identified transposons in the *Cen8s* were manually inspected and their orthologs defined by sequence alignment of the transposons and flanking orthologous genes. Because some regions of *Cen8s* from both Kasalath and *O. glaberrima* were not sequenced, and a unique 66.5-Kb fragment in *Cen8* of *O. glaberrima* had no orthologous sequence in the Nipponbare genome (Supplementary Figure 1), we only compared the sequences that clearly define orthologous regions in the other *Cen8s*.

We found that 367 transposons were shared between the *Cen8s* of Nipponbare and *O. glaberrima*, therefore these TEs likely existed in the ancestor of two rice species. Furthermore, 78 TEs, including 64 LTR retrotransposons, were present only in the *Cen8* of Nipponbare, suggesting that the transposition events likely occurred after divergence from their shared ancestor. Meanwhile, 33 TEs including 30 retrotransposons were detected only in *O. glaberrima* (Table 3) and these unshared transposons constituted ~10% of the *Cen8* sequence in *O. glaberrima*. Comparing the *Cen8s* of Kasalath and *O. glaberrima*, 291 TEs were shared and 29 and 80 TEs were present only in the *Cen8s* of *O. glaberrima* and Kasalath, respectively (Table 3). The polymorphic transposon rate between *Cen8s* of Nipponbare and *O. glaberrima* was 17.5% (78/445) in Nipponbare and 8.3% (33/400) in *O. glaberrima*, whereas, the rate between *Cen8s* of Kasalath and *O. glaberrima* was 21.6% (80/371) for Kasalath and 9.1% (29/320) for *O. glaberrima*, respectively. There was a higher transposition frequency in *Cen8s* of Nipponbare and Kasalath than in *O. glaberrima*—similar to the whole genomes comparison where more recent retrotranspositional activity was detected in the *O. sativa* lineage than in *O. glaberrima* (Wang et al., 2014).

**Table 3:** **Comparison of transposons in the orthologous regions of three *Cen8* sequences**.

**Genomes**		**Comparison of transposable elements**					
**Genome 1**	**Genome 2**	**Genome 1**			**Genome 2**		
		**Total**	**Shared**	**Unshared**	**Total**	**Shared**	**Unshared**
*O. glaberrima*	Nipponbare	400	367	33 (30)	445	367	78 (64)
*O. glaberrima*	Kasalath	320	291	29 (26)	371	291	80 (70)
Nipponbare	Kasalath	656	578	78 (48)	689	578	111 (89)

*Numbers in parentheses mean unshared retrotransposon numbers*.

**Figure 5:**
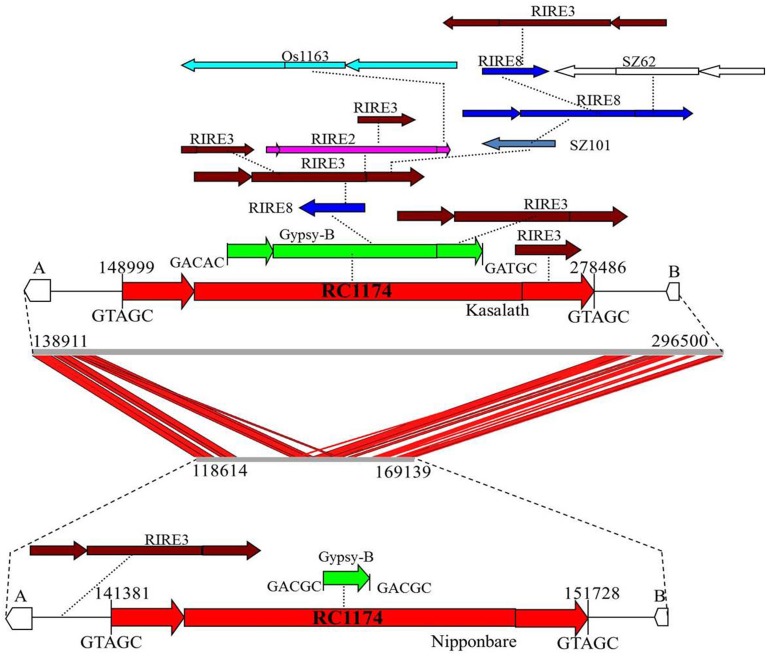
**Sequence alignment and transposon organization in one orthologous *Cen8* region**. Two genes, LOC_Os08g20020 (A) and LOC_Os08g20070 (B) are share among the *Cen8s* from Nipponbare and Kasalath. The middle is the sequence alignment between the 157.8-Kb sequence of Kasalath and the 50.5-Kb orthologous region from Nipponbare. Red lines represent shared sequences between two *Cen8s*. Top is transposon organization in *Cen8* of Kasalath where two host retrotransposons (RC1177 and Gypsy-B) were inserted by 13 unshared retroelements (not found in orthologous regions). Bottom is the orthologous transposons from *Cen8* of Nipponbare which has one unshared RIRE3 element and two shared host retroelements. The two host retroelements are flanked by 5-bp TSDs and TSDs for other retrotransposons are not shown.

Comparison between *Cen8s* of *O. sativa* revealed 78 and 111 unshared TEs in Nipponbare and Kasalath, respectively (Table 3). These 111 unique transposons covered ~600 kb or 27% of the *Cen8* of Kasalath. Previous comparisons between the *Cen8*s of Kasalath and Nipponbare indicated that nearly 33% of the sequence showed no colinearity with Nipponbare (Wu et al., 2009). Our data suggests that the unique sequence in Kasalath was due primarily to recent transpositions that interrupted colinearity. One exemplar is that between two orthologous genes, *LOC_Os08g20020* and *LOC_Os08g20070*, two retrotransposons, *RC1174* and *Gypsy-B*, had extensive insertions in Kasalath but not in Nipponbare. We identified a Nipponbare-specific *RIRE3* retrotransposon located between *LOC_Os08g20020* and *RC1174*, and 13 Kasalath-specific retrotransposons organized in a 130-Kb nested block and interrupting sequence colinearity (Figure 5).

## Discussion

### Rapid divergence of *CEN8s* in rice

Although the function of centromere is highly conserved among eukaryotes, centromeric repeats evolved rapidly and show little or no sequence similarity even between related species (Wong and Choo, 2004). For instance, the centromere satellite repeats exhibited no sequence similarity between two legumes, soybean (*G. max*) and common bean (*P. vulgaris*), (Gill et al., 2009; Iwata et al., 2013). In the cabbage family, centromeres of *B. rapa* consist of 176-bp satellite repeat (*CentBr*) and centromere retrotransposon (*CRB*, Ty1-copia group) which were completely different from that found in *A. thaliana*, 178-bp repeat *pAa* and *ATHILA* retroelement (Ty3-gypsy group) (Lim et al., 2007). We sequenced the *Cen8* from *O. brachyantha*, and identified five new LTR retrotransposons including *FRetro3* that was dominant in centromeric and pericentromeric regions of *O. brachyantha* (Gao et al., 2009). In *Cen8* of *O. granulata*, 10 novel LTR retrotransposon families were identified, and a single retrotransposon, *Gran3*, constituted nearly 43% of the centromeric sequences (Gao et al., 2011). It should be note that all new LTR retrotransposons, except centromere retrotransposon (*CR*) related elements, in *Cen8s* from *O. brachyantha* and *O. granulata* shared no sequence similarity between the two wild rice species or to cultivated rice. Together, these data indicated that centromere repeats have undergone rapid replacement and that centromere repeats, including the canonical *CRs*, have been overtaken by new retrotransposons and/or removed from centromere regions. However, most of previous studies focused on characterization of centromere specific repeats in more distantly related plants—last sharing a common ancestors more than 10 million years ago—and did not provide detailed comparisons of entire centromeres.

In this study, we compared comprehensively the *Cen8s* from two cultivated species. Our results confirmed the conservation and synteny of the centromere genes in the *Cen8* regions (Wu et al., 2009; Fan et al., 2011). However, we found that transposons have resulted in rapid and dramatic changes resulting in extensive sequence divergence. *Hopi* retroelements were present in both *O. sativa* and other wild species but not in *O. glaberrima* (Figure 1) suggesting that the *Hopi* family was removed from *O. glaberrima* lineage. This rapid decay of retrotransposon is similar to that observed in two wild rice species (Gao et al., 2009, 2011). However, we identified homologous sequences for most of the transposons between Nipponbare and Kasalath and *O. glaberrima* (Supplemental Table 1). Given that the split of the two cultivated rice species occurred ~1 million year ago, the time has probably not been long enough to replace entirely the centromeric transposon complement or for new elements to emerge. However, numerous recent transposition events were found that resulted in rapid sequence divergence of the centromere interrupting sequence colinearity (Figure 5). Taken together, these comparative analyses suggest another mechanism of rapid centromere evolution by which through massive and recent transpositions disrupting colinearity of the centromere. This is different from the replacement model of centromere transposons seen in comparisons of distantly related species.

### Impacts of transposons on functional centromere

Many reports have suggested an essential role for transposons in maintaining centromeric and telomeric stability and heterochromatic silencing (Maxwell et al., 2006; Zaratiegui et al., 2011). Additionally, transposons have been “domesticated” for service in various cellular and biochemical processes. For instance, the orthologs of a human centromere-binding protein (CENP-B), Cbp1 proteins, likely evolved from a domesticated Pogo-like DNA transposase (Casola et al., 2008) and were associated with the RNAi-mediated transposon silencing and the eradication of retrotransposons in fission yeast genomes (Cam et al., 2008). We found that ~70% of the *Cen8s* from two cultivated rice species was occupied by transposons, this fraction is much higher than that the whole genomes (International Rice Genome Sequencing Project, 2005; Wang et al., 2014) highlighting the highly repetitive nature of centromeres. Even though the majority of TEs in the *Cen8s* were located in between genes some TEs were located in centromere genes, serving as CDSs of expressed genes (Table 2). Comparisons of gene-associated TEs indicated that some TEs inserted recently into centromere genes (Supplemental Figure 2). Thus, transposons may be involved in the divergence of the centromere genes.

Despite the dramatic sequence diversity of centromeres from various eukaryotic organisms, the histone H3-related protein (CENH3), which replaces canonical histone H3 in the nucleosomes of functional centromeres, is highly conserved and can interact with all centromeric DNAs studied so far (Henikoff et al., 2001). Genetic and biochemical analysis revealed that CENH3 is an essential component for the assembly of a functional kinetochore during cell division (Wieland et al., 2004), and can be used as a biochemical marker to determine the positions of functional centromeres. Previous results from chromatin immunoprecipitation (ChIP) with anti-CENH3 antibody suggested that the *CR* elements and centromere satellites likely bind CENH3 and participate in centromeric localization (Zhong et al., 2002; Nagaki et al., 2004). However, it is not clear if other sequences in centromere can interact with CENH3. We searched rice CENH3 immunoprecipitated DNA sequences (Zhang et al., 2013) and found that 165 TEs families showed significant sequence similarity with the ChIP sequence data. Some TEs were targeted by numerous reads and reads related to *RIRE3* and *RIRE8* were even more abundant than *CRR*-related sequences (Supplemental Table 3). Both retrotransposons are dispersed throughout the rice genome, however, a higher density was detected in centromeric and pericentromereic regions (Figure 4). Given that *RIRE3* and *RIRE8* were dominant in *Cen8s*, we hypothesize that these two retrotransposons are likely involved in organization of functional centromeres in rice. The identification of CENH3-associated DNA transposons also suggests that DNA transposons may be important for recognization of the centromere binding protein and assembly of specific centromere structures.

### Nested organization of transposons in *CEN8s*

Nested organizations of LTR-retrotransposons has been frequently observed in genomes with high transposon densities (Kronmiller and Wise, 2008). However, they are not common in euchromatic regions of rice (Du et al., 2006; Kronmiller and Wise, 2008). For example, we analyzed the 19.4-Mb short arm of chromosome 3 (*Chr3s*) of Nipponbare (Roulin et al., 2010), and identified 45 nested blocks of transposons ranging in size from 1380 to 124,988 bp (15,467 bp average), contributing 3.6% of the *Chr3* short arm sequence. However, 57 nested TE blocks were identified in the *Cen8* of Nipponbare that accounted for 39.1% of the *Cen8*. The density of nested TE block in *Cen8* was 23.8 blocks/Mb (57/2.4 Mb), much higher than in *Chr3s* with 2.3 blocks/Mb (45/19.4 Mb). These data indicated that the TEs in *Cen8* exhibited higher density and more complex organization patterns than in euchromatic regions of rice, more similar to maize (Kronmiller and Wise, 2008).

Nested organization of transposons may have important impacts on centromere evolution and on the host genome. First, TE insertions into transposons may be less harmful for host genome as transposons insertions can be harmful or lethal if inserted into important genes. Thus, the transposons frequently integrate into gene poor regions. For instance, massive transposition events were detected in the *ddm1* mutant of *A. thaliana* and most of the active TEs inserted in centromeric repeats (Tsukahara et al., 2009). Second, nested insertions of transposons likely represent an important way to silence transposons as the activity of transposons can lead to meiotic failure and lagging chromosomes (Volpe et al., 2002; Bourchis and Bestor, 2004). We identified nested transposons in both terminal repeats and internal regions of host transposons. Some nested TEs, such as MITEs, are small and may not affect the mobility of host TE, however, many nested TEs were large LTR retrotransposons that likely interrupted transcription and transposition activity of host TEs. Third, nested insertions resulted in rapid diversification of the *Cen8s* and appeared to have played an important role in reshaping these centromeres. The nested transposon blocks contribute more than half of centromere transposons in the *Cen8s* from both cultivated rice species, and we identified many unshared TEs in the nested blocks that both disrupt sequence colinearity and increase sequence centromere divergence, in short time period. Lastly, nested TEs provide useful information to study *Cen8* evolution. Nested transposons offer valuable information to track the evolutionary history of transposons and to reconstruct ancient TE insertions relative to their “pre-nested” states (Kronmiller and Wise, 2008). The comparative analysis of nested transposons between three *Cen8s* allowed us to identify the transposons that likely existed in the *Cen8* region before the divergence of two cultivated rice species. We found a number of accession-specific transposons and that many insertions in the nested TEs were recent and greatly expanded the *Cen8* sequences (Figures 2, 5).

### Conflict of interest statement

The authors declare that the research was conducted in the absence of any commercial or financial relationships that could be construed as a potential conflict of interest.

## Supplementary material

Supplementary Figure 1 **The three *Cen8*s and their corresponding locations in the chromosome 8 pseudomolecule (GenBank accession number: AP008214)**. Black boxes and red box in *Cen8* of *O. glaberrima* represent two inversions and unique sequences, respectively, blue boxes in *Cen8*s of Nipponbare and Kasalath are sequence that are absent in Cen8 of *O. glaberrima*.

Supplementary Figure 2 **The structure of an expressed centromere gene *LOC_Os08g221590***. The gene model was supported by the full-length cDNA (AK111577), the orange and white box mean exon and UTR of the gene, respectively. The gene in *Cen8s* of Nipponbare and *O. glaberrima* harbors four shared transposons: a Mutator element Os1617, two MITEs Os0004, and Os0214, and a truncated solo LTR of Os0404. The gene in Kasalath's *Cen8* has four shared transposons and two new insertions from Os0015 (MITE) and Os0229.
