# Supplementary material for: Transposons play an important role in the evolution and diversification of centromeres among closely related species
Source: Front Plant Sci. 2015 Apr 7;6:216. doi: 10.3389/fpls.2015.00216 (PMC4387472; doi:10.3389/fpls.2015.00216)
Supplement: Supplementary Figure 1 — The three Cen8s and their corresponding locations in the chromosome 8 pseudomolecule (GenBank accession number: AP008214). Black boxes and red box in Cen8 of O. glaberrima represent two inversions and unique sequences, respectively, blue boxes in Cen8s of Nipponbare and Kasalath are sequence that are absent in Cen8 of O. glaberrima. [file Presentation1.ZIP › Table 2_v2.DOCX]

Supplementary Table 2. Summary of nested transposons in 3 *Cen8*s

| Type of TE insertion | | Nipponbare | | *O. glaberrima* | | Kasalath | |
| --- | --- | --- | --- | --- | --- | --- | --- |
|  |  | Copy  No. | Size  (bp) | Copy  No. | Size  (bp) | Copy  No. | Size  (bp) |
| RNA into RNA | element | 38 | 370,064 | 16 | 156,177 | 46 | 454,653 |
|  | solo-LTR | 31 | 90,561 | 24 | 72,067 | 32 | 96,239 |
|  | fragment | 30 | 112,734 | 31 | 103,926 | 29 | 117,080 |
|  | all | 99 | 573,359 | 71 | 332,170 | 107 | 667,972 |
|  |  |  |  |  |  |  |  |
| RNA into DNA | element | 2 | 23,644 | 1 | 3,873 | 0 | 0 |
|  | solo-LTR | 5 | 9,014 | 5 | 8,104 | 4 | 11,412 |
|  | fragment | 0 | 0 | 0 | 0 | 1 | 7,364 |
|  | all | 7 | 32,658 | 6 | 11,977 | 5 | 18,776 |
|  |  |  |  |  |  |  |  |
| DNA into RNA | element | 11 | 19,147 | 3 | 2,601 | 5 | 9,941 |
|  | fragment | 8 | 2,476 | 9 | 3,163 | 9 | 4,549 |
|  | all | 19 | 21,623 | 12 | 5,764 | 14 | 14,490 |
|  |  |  |  |  |  |  |  |
| DNA into DNA | element | 14 | 10,021 | 4 | 8,465 | 11 | 13,808 |
|  | fragment | 3 | 1,327 | 0 | 0 | 4 | 644 |
|  | all | 17 | 11,348 | 4 | 8,465 | 15 | 14,452 |
|  |  |  |  |  |  |  |  |
| Total nested TEs | element* | 65 | 422,876 | 24 | 171,116 | 62 | 478,402 |
|  | solo-LTR | 36 | 99,575 | 29 | 80,171 | 36 | 107,651 |
|  | fragment* | 41 | 116,537 | 40 | 107,089 | 43 | 129,637 |
|  | all | 142 | 638,988 | 93 | 358,376 | 141 | 715,690 |

Note: * Total nested element and fragment from both DNA and RNA TEs.
